# Co-expression of stress-responsive regulatory genes, *MuNAC4, MuWRKY3* and *MuMYB96* associated with resistant-traits improves drought adaptation in transgenic groundnut (*Arachis hypogaea* l.) plants

**DOI:** 10.3389/fpls.2022.1055851

**Published:** 2022-11-16

**Authors:** Boya Venkatesh, Amaranatha R. Vennapusa, Nulu Jagadeesh Kumar, N. Jayamma, B. Manohara Reddy, A. M. Anthony Johnson, K. V. Madhusudan, Merum Pandurangaiah, K. Kiranmai, Chinta Sudhakar

**Affiliations:** ^1^ Plant Molecular Biology Laboratory, Department of Botany, Sri Krishnadevaraya University, Anantapuram, India; ^2^ Department of Agriculture and Natural Resources, Delaware State University, Dover, DE, United States; ^3^ Department of Botany, Government College (Autonomous), Anantapuram, India; ^4^ Department of Biotechnology, St. Josephs University, Bengaluru, India; ^5^ Department of Botany, Government College, Cluster University, Kurnool, India

**Keywords:** groundnut, drought stress, transcription factor, roots, multigene transgenics, water use efficiency

## Abstract

Groundnut, cultivated under rain-fed conditions is prone to yield losses due to intermittent drought stress. Drought tolerance is a complex phenomenon and multiple gene expression required to maintain the cellular tolerance. Transcription factors (TFs) regulate many functional genes involved in tolerance mechanisms. In this study, three stress-responsive regulatory TFs cloned from horse gram, (*Macrotyloma uniflorum* (Lam) Verdc.), *MuMYB96*, involved in cuticular wax biosynthesis; *MuWRKY3*, associated with anti-oxidant defense mechanism and *MuNAC4*, tangled with lateral root development were simultaneously expressed to enhance drought stress resistance in groundnut (*Arachis hypogaea* L.). The multigene transgenic groundnut lines showed reduced ROS production, membrane damage, and increased superoxide dismutase (SOD) and ascorbate peroxidase (APX) enzyme activity, evidencing improved antioxidative defense mechanisms under drought stress. Multigene transgenic plants showed lower proline content, increased soluble sugars, epicuticular wax content and higher relative water content suggesting higher maintenance of tissue water status compared to wildype and mock plants. The scanning electron microscopy (SEM) analysis showed a substantial increase in deposition of cuticular waxes and variation in stomatal number in multigene transgenic lines compared to wild type and mock plants. The multigene transgenic plants showed increased growth of lateral roots, chlorophyll content, and stay-green nature in drought stress compared to wild type and mock plants. Expression analysis of transgenes, *MuMYB96*, *MuWRKY3*, and *MuNAC4* and their downstream target genes, *KCS6*, *KCR1*, *APX3*, *CSD1*, *LBD16* and *DBP* using qRT-PCR showed a two- to four-fold increase in transcript levels in multigene transgenic groundnut plants over wild type and mock plants under drought stress. Our study demonstrate that introducing multiple genes with simultaneous expression of genes is a viable option to improve stress tolerance and productivity under drought stress.

## Introduction

Drought, the detrimental abiotic stress, majorly affects the productivity of rain-fed crops and results in yield losses. Groundnut (*Arachis hypogaea* L.) is one of the major oil seed crops with a worldwide production of ~48.75 million metric tons cultivated under ~34.10 million hectares. Nearly 2/3rd of its production was used for oil production (FAOSTAT, 2019). As a rain-fed crop, groundnut is more prone to periodic drought stress, and significant effects on plant physiological processes were reported both in vegetative and reproductive phases (Kambiranda et al., 2011; Farooq et al., 2012; Jongrungklang et al., 2013). Drought tolerance is a complex phenomenon accomplished by the multiple traits at morphological, cellular, and molecular levels. To substantiate the adverse effects of drought stress, plants have adopted multiple drought tolerance traits such as cellular level tolerance, reduced transpirational water loss, improved water mining, and conservation traits, which are controlled either directly or indirectly by regulatory and/or functional genes (Mickelbart et al., 2015; Xiao et al., 2017). Transcription factors (TFs) act as molecular switches by regulating the expression of downstream genes by binding to the cis-acting elements at the promoter region of the genes (Franco-Zorrilla et al., 2014). MYB (Myeloblastosis), WRKY, and NAC (NAM, ATAF, and CUC) are the three large TF families in the plant kingdom and are involved in diverse developmental and stress tolerance mechanisms (Guo et al., 2013; Hrmova and Hussain, 2021; Manna et al., 2021). Overexpression of TF genes through genetic engineering was reported as a viable option to integrate stress adaptive/stress tolerance traits and confer tolerance against various abiotic stresses, including drought in crop plants (Yamaguchi-Shinozaki and Shinozaki, 2006; Buscaill and Rivas, 2014; Shao et al., 2015; Erpen et al., 2018).

The architecture and distribution of the root system is the key feature of water mining traits (de Dorlodot et al., 2007; Coudert et al., 2010) and determines plants’ ability to acquire water and nutrients from the soil to maintain plant growth under drought conditions (Lynch, 1995). Root perceives water scarcity and allows plants to adapt to drought stress by increasing their length, density, and volume (Hammer et al., 2009; Bengough et al., 2011; Anjum et al., 2017). Improving root traits through the genetic engineering approach conferred enhanced drought tolerance in most agricultural crops (Hodge et al., 2009). Overexpression of TFs like NAC1, ERF48, Alfin1, DREB2A, etc. has been involved in root growth and development under moisture stress (Janiak et al., 2016; Ramu et al., 2016; Jung et al., 2017). NAC4, TF with a characteristic NAC domain, was reported to induce lateral root growth under water-limited conditions through auxin signaling in an ABA-dependent manner. Overexpression of the *NAC4* gene shows increased root length and lateral roots and enhanced drought tolerance in transgenic groundnut plants (Pandurangaiah et al., 2014). Root growth was stimulated under osmotic stress in transgenic Arabidopsis lines upon overexpression of the *TaNAC4-3A* gene and showed an improved drought tolerance (Mei et al., 2021).

Conservation of tissue water status was a great challenge for the plants during limited water conditions. Plants maintain relatively high tissue water content by minimizing water loss through increased cellular level tolerance (CLT), reduced cellular damage, and evapotranspiration. Osmolytes like proline, soluble sugars, and quaternary ammonium compounds like betaines. etc., will be produced in higher concentration inside the cell and maintains cell turgor and water potential during drought stress (Varshney et al., 2011; Fang and Xiong, 2015; Nahar et al., 2017; Yao et al., 2021). Overexpression of TF genes, *DREB*, *NAC*, *MYB*, *WRKY*, and *bZIPs*, etc., in groundnut and other plants showed enhanced osmolyte accumulation and antioxidative defense systems along with other physio-biochemical traits conferring tolerance to different abiotic stresses, including drought through cellular level tolerance (Bhatnagar-Mathur et al., 2007; Pruthvi et al., 2014; Joshi et al., 2016; Kiranmai et al., 2016; Sarkar et al., 2016; Kishor et al., 2018; Manna et al., 2021). A WRKY transcription factor, WRKY3, belonging to group-I WRKY TFs, has been reported to induce resistance against several biotic stressors such as pathogens bacteria and fungi, etc. (Lai et al., 2008; Şahin-Çevik et al., 2014; Guo et al., 2018) and herbivory (Skibbe et al., 2008). In addition to biotic stresses, WRKY3 TF is also reported to be involved in cellular level tolerance mechanisms against different abiotic stresses, including salt, cold, and drought (Liu et al., 2013; Kiranmai et al., 2016; Hichri et al., 2017).

Along with CLT, anatomical traits such as stomata, cuticular wax content, etc., help plants to conserve tissue water under moisture stress (Gomes and Prado, 2007). Cuticle serves as an indispensable barrier and protects the plants from harmful radiations (UV-B), evapotranspiration water loss and also has a positive effect on water use efficiency during water-limited conditions (Jenks et al., 2001; Lee and Suh, 2015; Fich et al., 2016; Iqbal et al., 2020). Biosynthesis and deposition of cuticular waxes in response to drought stress were genetically controlled (Riederer and Schreiber, 2001; Nawrath, 2006) by several TF genes such as *WAX1, MYB96, MYB94, WIN1/SHN1, WXP1, WR1, AP2/EREBP, DWA1*, and functional genes, *KCS1, CER1*, and *FAR1*, etc., were reported to be conferring resistance in several crop plants (Seo and Park, 2011; Xue et al., 2017; Lewandowska et al., 2020). MYB96, an R2R3-MYB TF characterized by two MYB domain repeats, is reported to positively regulate biosynthesis and deposition of cuticular waxes on aerial plant organs and increased drought resistance upon overexpression (Seo et al., 2009; Seo et al., 2011; Lee et al., 2014; Lee et al., 2016).

Introducing multiple genes contributing to different traits with simultaneous expression in a single construct is a reliable and time-saving approach (Goel and Singh, 2018; Vennapusa et al., 2022). Furthermore, co-expression of multiple genes in plants has been shown to improve the tolerance against different abiotic stresses compared to single gene transgenics (Singla-Pareek et al., 2003; Babitha et al., 2013; Nguyen et al., 2013; Augustine et al., 2015; Parvathi et al., 2015) including groundnut (Pruthvi et al., 2014; Ramu et al., 2016).

Horsegram is a potential dryland legume crop for future and is source of mining genes for abiotic stress tolerance, as this crop is well suited for cultivation in very poor soils under receding moisture level in drought prone areas, saline soils and high temperature regions (Reddy et al., 2008; Pandurangaiah et al., 2014; Kiranmai et al., 2018) In the present study, three transcription factor genes, *MuMYB96, MuWRKY3*, and *MuNAC4*, involved in improving the water conservation, cellular level tolerance, and root traits cloned in a single cassette through modified gateway cloning technology, and transferred to groundnut for developing the multigene transgenic plants for improved drought stress tolerance.

## Materials and methods

### Plant material, growth conditions and stress treatments

Seeds of horsegram (*Macrotyloma uniflorum* (Lam) Verdc.) cultivar VZM1 and groundnut (*Arachis hypogaea* L.) cultivar K-6 were procured from Regional Agricultural Station, Rekulakunta and Kadiri, Anantapuram, respectively. Seeds were sown in earthen pots containing soil and farmyard manure in a 3:1 proportion maintained in the departmental botanical garden under natural photoperiod (10–12 h; 27 ± 4 °C). After 30 days post-sowing, drought stress was induced by withholding water to one set of pots, and respective fully watered controls were maintained in another set of pots. Ten days after stress imposition, fully opened fresh leaf samples were collected, pooled, flash frozen in liquid nitrogen, for futher analysis.

### Isolation of genes

Total RNA was isolated from stress-adapted horse gram leaves subjected to drought stress using the Trizol reagent (Invitrogen). The leaf material (100mg) from drought stressed horsegram plants was ground to amorphous powder using liquid nitrogen and added with 1ml of Trizol reagent containing guanidium thiocyanate (Simms et al., 1993). The supernatant was separated after centrifugation and nucleic acids portion was aspired using chloroform. The RNA was precipitated with isopropanol and sodium citrate/NaCl (1:1) solution. The RNA precipitate washed with ethanol, dried and dissolved in sterile diethylpyrocarbonate (DEPC) water and the same was used as a template for cDNA synthesis using the RevertAid reverse transcriptase enzyme (Thermo Scientific, USA).

Individual gene-specific primers were used to isolate individual genes. The PCR setup and annealing temperatures were optimized for all three genes *MuMYB96, MuWRKY3*, and *MuNAC4*, individually to get the specific gene amplification in the gradient thermal cycler (Eppendorf, Hamburg). PCR was initiated by a hot start at 94 ˚C for 5 min followed by 30 cycles of 94 ˚C for 1 min, 59.1 ˚C (*MuMYB96*), 58.2 ˚C (*MuWRKY3*) and 53.1 ˚C (*MuNAC4*) for 45 s and 72 ˚C for 1 min with a final extension of 10 min. and the list of primer sets were given in **Supplementary Table 1**. The amplification was checked on 0.8% agarose gel. The authenticity of the PCR product was checked by restriction enzyme digestion, confirmed and cloned into a T/A vector (Thermo Scientific, USA) and sequenced.

### Development of gene cassettes and gateway entry vectors

Each gene was cloned under the specific promoter by conventional restriction digestion and ligation strategy to develop gene expression cassettes. *MuMYB96* gene was cloned under *rbcs* promoter and terminator in the Impact vector (IM1.1) (P_rbcs_: *MuMYB96*:T_rbcs_), *MuWRKY3* gene was cloned into the pRT100 vector under CaMV2x35S promoter and a polyA tail terminator (P_CaMV2x35S_: *MuWRKY3*:T_polyA_) and *MuNAC4* gene expression cassette were developed using a pB4NU plasmid vector carrying ubiquitin promoter and *nos* terminator (P_Ubi_: *MuNAC4*:T_nos_) using specific restriction enzymes. Then, these three gene cassettes were sub-cloned into modified gateway entry vectors. The *MuMYB96* gene cassette was released by digesting with *HindIII* and *PacI* and was ligated to the linearized pGATE L1-L4 entry vector, and the *MuWRKY3* gene construct was sub-cloned into pGATE R4-R3 using *SphI* enzyme. Finally, the *MuNAC4* expression cassette was excised from the pB4NU vector and introduced into the pGATE L3-L2 vector between *EcoRI* and *HindIII* restriction sites to prepare the entry clones.

### Construction of multigene cassette

The three genes were stacked together in a plant expression binary vector by recombination reaction. Three gateway entry vectors, *pGATEL1L4-P_rbcs_ : MuMYB96:T_rbcs_*, *pGATE R4R3-P_CaMV2x35S_:MuWRKY3:T_polyA,_* and *pGATEL3L2-P_Ubi_ : MuNAC4:T_nos_* were allowed to a recombination reaction with destination vector, pKM12GW containing a neomycin phosphotransferase (*nptII*) gene as plant selectable marker in 1:1:1:3 ratio respectively. The reaction was carried out at 25°C overnight in the presence of LR clonase enzyme, and proteinase K was used to terminate the reaction (Vemanna et al., 2013). The resulting recombinant vector was used to transform *Agrobacterium tumefaceins*.

### Transformation of multigene construct into groundnut

The binary vector expressing *pKM12GW*-*MuMYB96*:*MuWRKY3*:*MuNAC4* was transferred into *Agrobacterium tumefaciens* EHA105 strain by Freeze-thaw method, and colony PCR was carried out to identify positive transformants (Weigel and Glazabrook, 2006). A tissue culture-independent agrobacterium mediated *in planta* transformation protocol (Rohini and Rao, 2001) was adopted to develop the groundnut transgenics. Two-day-old germinating groundnut (cultivar variety K6) sprouts were pricked at the embryonic site and co-cultivated with agrobacterium culture for 16 hours at 28°C with gentle agitation, followed by rinsing with cefotaxime (500μg/ml) for 2 minutes and later with sterile distilled water. The seedlings were acclimatized in a plant growth chamber on a sterilized soilrite at controlled conditions (28 ± 2°C; 16 hours of light/day; light intensity-400-550 μE/m^2^/s) RH- 60%). Another set of seeds were transformed with *Agrobacterium* cells carrying *pKM12GW* vector without transgenes and were treated as mock plants. After acclimatization, plantlets were transferred to a greenhouse, maintained at 28°C, and allowed to grow under natural photoperiodic conditions till harvest. Putative transgenic groundnut plants were identified by kanamycin screening and PCR analysis using npt-II primers. The seeds from the T_0_ generation were germinated on MS agar medium (Himedia, Mumbai, India) supplemented with 200mg/L of kanamycin under controlled environment chambers (Conviron A1000, Canada). The seedlings showing normal shoot and root growth were transferred to sterile soilrite for acclimatization, then transferred to the earthen pots and maintained in the greenhouse. Integration of all three genes in genomic DNA was confirmed by PCR using gene-specific primers. Transgenic plants showing the integration of three genes were considered positive transgenic plants and advanced to the subsequent generations.

### Expression analysis of transgenes by qRT-PCR

The expression of transgenes, *MuMYB96, MuWRKY3*, and *MuNAC4* was analyzed in putative T_3_ transgenic groundnut plants and wild type, subjected to drought stress for 10 days. Fully opened leaf samples were used for analysis. Total RNA isolated from leaf samples was treated with a Turbo DNase treatment kit (Thermo Fisher Scientific, USA) as per the manufacturer’s protocol to remove any DNA traces. cDNA was synthesized using Revert Aid M-MuLV Reverse Transcriptase (Thermo Fisher Scientific) as per the manufacturer’s instructions. qRT-PCR mix was comprised of 1× using Power SYBR Green Master Mix (Ambion, USA), 20 ng of cDNA, and 0.2 µM of forward and reverse primers. The housekeeping gene, *actin*, was used as an internal control in the reaction. The RT-PCR analysis was done on Applied Biosystems Step One Real-Time PCR machine with standard cycling comprising 95°C for 30 s, 40 cycles of 95°C for 1 s, 60°C for 20 s, and a melt curve analysis. Relative quantification was studied using 2−^Δ Δ^ CT method (Livak and Schmittgen, 2001).

In addition to transgenes, a few downstream genes such as *KCS6*, *KCR1, APX3, CSD, LBD16* and *DBP* were also analyzed using qRT-PCR. The gene sequences of the selected down-stream genes were obtained from genome of *Arachis hypogaea* (http://peanutbase.org/home) and the same sequences were used to design primers using Primer Express™ Software v3.0.1. Each gene was analyzed in three biological samples, and three reaction replicates were performed for each biological sample. The primers used for PCR analysis were given in **Supplementary Table 2**.

### Scanning electron microscopy

Scanning Electron Microscopy (SEM) was employed to investigate the cuticular wax depositions and stomatal structure on the leaf surface of transgenic, mock, and wild type groundnut plants subjected to drought stress. First, the freshly harvested leaf bits of 1cm^2^ were vacuum dried and mounted onto aluminum stubs, followed by gold nanoparticle coating with a fully automated vacuum sputter smart coater (DII-29030SCTR, JOEL, USA). Then, leaf-mounted stubs were transferred to the scanning electron microscope (JOEL JSM-IT500, Japan) to visualize the extent of epicuticular wax depositions on the leaf surface (Lokesh et al., 2019).

### Evaluation of transgenic groundnut plants for drought stress tolerance

Physiological and biochemical parameters related to cellular level tolerance and WUE were carried out under drought stress in putative transgenic groundnut lines along with mock and wild type plants. The drought stress was imposed on thirty-day-old plants by withholding irrigation for ten days and fully opened leaf samples were collected uniformly from each set of plants. Three biological samples, and three reaction replicates were performed for each physiological and biochemical assay.

### Relative water content

Relative water content (RWC) was measured in multigene transgenic plants along with wild type plants under drought stress conditions. First, leaf discs were prepared from matured leaves, and fresh weight was measured. Then the leaf discs were immersed in sterile water for four hours, and the weight was recorded as turgid weight; then, the leaf discs were dried in a hot air oven for 48 h, and the dry weight was determined. Finally, RWC was calculated using the formula (Vemanna et al., 2017).

### Total chlorophyll content

Chlorophyll pigments were extracted from drought-stressed leaves of multigene transgenics and wild type plants by boiling them in dimethyl sulfoxide (DMSO) at 65°C for 10 min. The extracted chlorophylls were read at 645nm and 663nm using a spectrophotometer (Shimadzu UV 1800, Japan). The total chlorophyll content was estimated according to Hiscox and Israelstam (1979) and expressed as mg/g F.W (Vennapusa et al., 2022).

### Epicuticular wax content

Epicuticular waxes on the leaf surface were separated and quantified according to the method given by Mamrutha et al. (2010). The waxes were extracted with chloroform from the leaf surface and treated with acidic-potassium dichromate (K_2_Cr_2_O_7_) to give a coloured compound. The ECW content was calculated using a colorimetric method and expressed as µg/g F.W.

### Total soluble sugars (TSS) and proline content

Total soluble sugar content was determined following Kiranmai et al. (2018). Water extract of leaf was treated with 5% phenol and 98% sulphuric acid and incubated at room temperature for 1 hr, and the absorbance was measured at 485nm. The TSS content was expressed as µg/g F.W.

Accumulation of proline content in the leaf samples was determined as described by Bates et al. (1973). Leaf extract was prepared in 3% sulphosalicylic acid, heated, treated with acid ninhydrin and acetic acid, and incubated at 100°C for 1hr. The reaction was terminated on ice, and the chromophore was extracted with 4 mL toluene and mixed thoroughly. The toluene phase was separated and measured with a spectrophotometer 540nm using toluene as blank, and the proline content was calculated from the standard curve and expressed as µmol/g F.W (Vemanna et al., 2017).

### Lipid peroxidation

The extent of cell membrane damage was calculated indirectly by measuring the malondialdehyde content, a product of lipid peroxidation of membrane lipids. The leaf material from drought stressed transgenic, mock and wild type plants was used to estimate thiobarbituric acid, a reactive compound of malondialdehyde, and calculated against a standard MDA graph (Nisarga et al., 2017).

### ROS and scavenging system

Superoxide ion and hydrogen peroxide contents were quantified in the transgenic groundnut plants, wild type and mock plants exposed to drought stress. Superoxide ions were estimated by a colorimetric method according to Kiranmai et al. (2018) by treating with nitroblue tetrazolium (NBT) solution, and hydrogen peroxide content was measured as described by Junglee et al. (2014) and Nareshkumar et al. (2015).

The efficacy of the anti-oxidant defense system was analyzed by measuring the activity of superoxide dismutase (SOD) (Kiranmai et al., 2018) and ascorbate peroxidase (APX) (Vemanna et al., 2017) in the leaves of multigene transgenic and wild type plants using a colorimetric method.

### Growth and yield attributes

After harvesting, morphological and yield traits such as shoot length, root length, shoot dry weight, root dry weight, number of pegs, number of pods, and dry weight of pods were measured for transgenic plants along with the wild type and mock plants (Vemanna et al., 2017).

Briefly, multigene transgenic groundnut lines, wild type and mock plants were gently uprooted from the pots. Roots were cleaned using tap water to remove debris and soil particles properly and maximum care was taken to avoid the loss of roots. Number of pegs and pods were recorded. Shoot and root parts were separated and their length was recorded using an ordinary ruler. Then the shoot, root parts and pods were dried at 50°C for 48 hours in a hot air oven and dry weight was recorded using digital scale. Data was recoded in three biological sets with triplicates and the results were shown mean-values per plant.

### Statistical analysis

All the physiological and biochemical experiments were conducted in three biologically independent experiments, statistical analyses were performed using R version 4.2.0, and ANOVA was performed using the R package agricolae with Fisher’s LSD test to separate means and significance at P ≤ 0.05 (de Mendiburu, 2014; Pandian et al., 2020). Data presented are mean values and standard error ( ± SE).

## Results

### Development of multigene expressing transgenics groundnut plants

**Figure 1:**
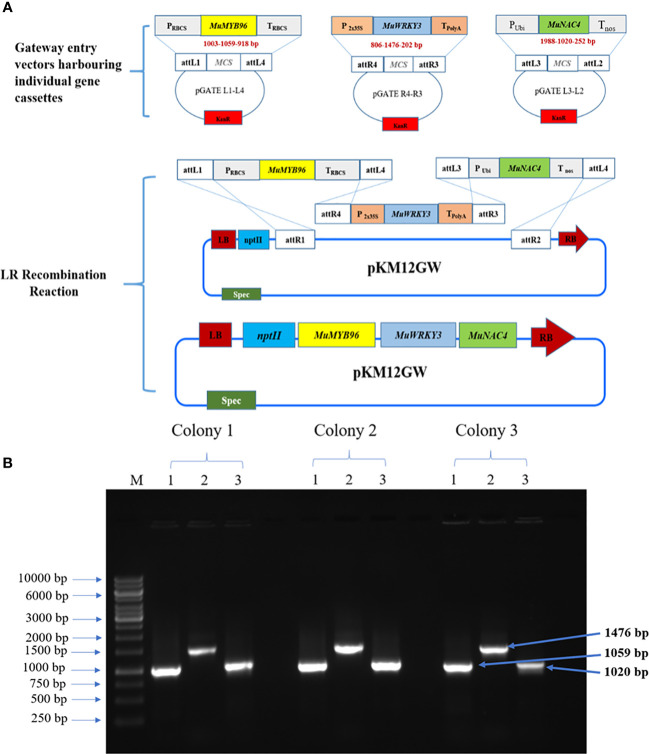
Development of multigene cassette harboring *MuMYB96, MuWRKY3* and *MuNAC4* genes: **(A)**. Over view of steps involved in the development of multigene construct through modified gateway cloning technology. **(B)** PCR confirmation of *MuMYB96* (1059 bp), *MuWRKY3* (1476 bp) and *MuNAC4* (1020 bp) in *Agrobacterium tumefaciens*. Lane M: DNA ladder, Lane 1,2,3: positive colony.

**Figure 2:**
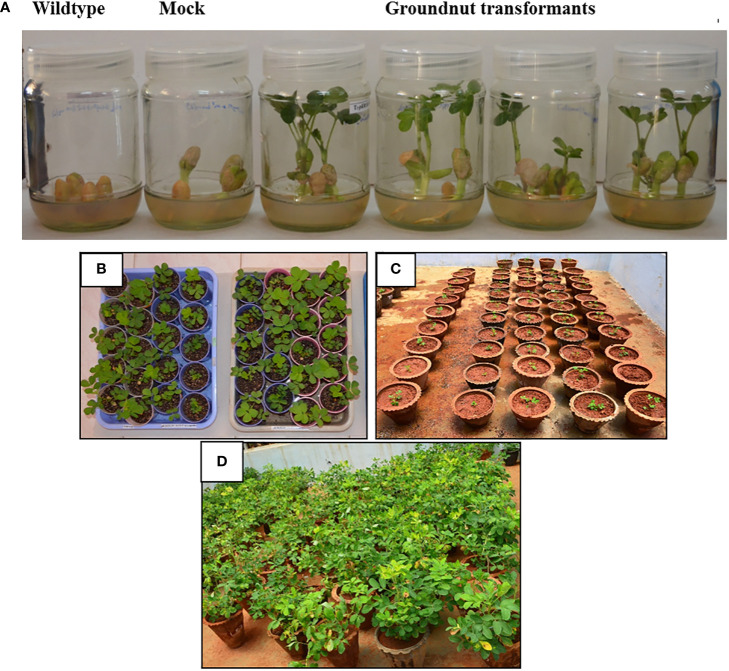
Screening of putative transformants on selection media and advancement: **(A)**. Selection of groundnut transformants on Kanamycin screening. Wild type seeds showing inhibited germination on kanamycin containing MS half strength medium. Mock plant (groundnut plants transformed with empty vector) seeds showing the delayed germination on kanamycin containing MS half strength medium. Multigene (*pKM-MuMYB96:MuWRKY3:MuNAC4*) transgenic groundnut plants showing normal growth on the kanamycin medium. Different stages of growth and development of T_3_ transgenic groundnut plants. **(B)**. Plants growing on soil-rite in a plant-growth chamber after kanamycin screening. **(C)** Acclimatization of plants on pot culture containing soil manure mixture (3:1) in green house. **(D)** 90-day-old plants in the green house under natural photoperiodic condition.

The three TF genes, *MuMYB96, MuWRKY3*, and *MuNAC4* were amplified from cDNA synthesized from horse gram leaf RNA samples **(** **Supplementary Figure 1** **)** and sequence (**Supplementary Table 3**). All the three genes, were sub cloned to pRT100 vectors under CaMV35S promoter and polyA terminator at Apa1 and Nco1, Kpn1 and Nco1, and Kpn1 and BamH1 sites, respectively **(** **Supplementary Figure 1A** **)**. The individual gene cassettes, *P_rbcs_: MuMYB96:T_rbcs_ P_CaMV2x35S_:MuWRKY3:T_polyA_* and *P_Ubi_ : MuNAC4:T_nos_* were stacked into a single multigene construct in a plant binary vector, *pKM12GW* through the LR clonase reaction using a modified multisite gateway cloning technology (Vemanna et al., 2013). The plant destination vector carrying all the three genes, *pKM-MuMYB96:MuWRKY3:MuNAC4* in *Agrobacterium* (**Figures 1A, B** **)**, was transferred to groundnut seedlings. Putative transgenic groundnut lines were selected on a kanamycin medium. The putative multigene transgenic groundnut plants showed growth on the kanamycin selection medium, whereas the wild type plants failed to germinate or showed stunted growth. Transgenic plants that showed normal growth were acclimatized in the greenhouse, maintained till harvest, and/or advanced to the next generation **(** **Figure 2**). Integration of all three transgenes was confirmed in putative transgenic plants by PCR analysis using genomic DNA as a template (**Supplementary Figure 2**). The multigene transgenic groundnut plants showing kanamycin resistance and gene integration were advanced to the next generation and the transgenic events were shown in **Supplementary Figure 3**.

### Expression of transgenes and stress responsive genes in multigene transgenic groundnut plants under drought stress

**Figure 3:**
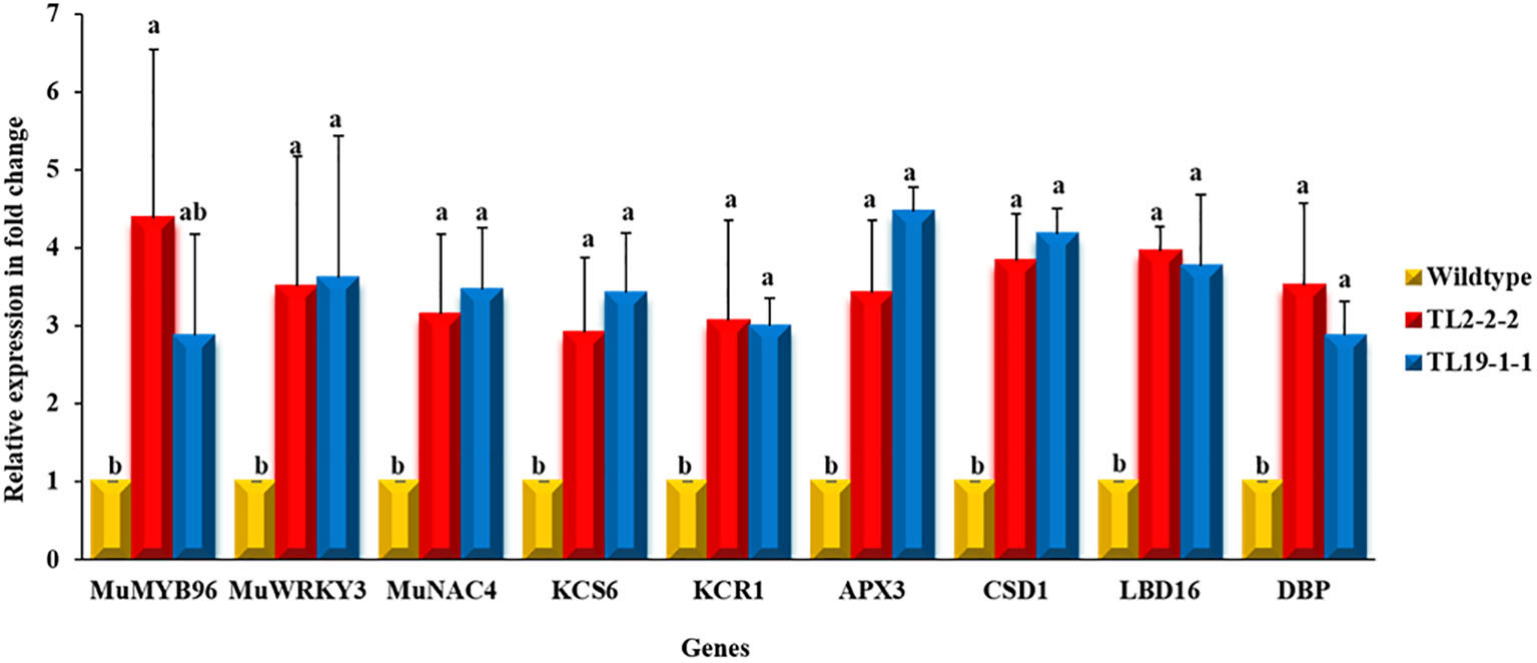
Expression profiling of transgenes and downstream genes using qRT-PCR: The leaf samples of multigene groundnut transgenic plants and wild type subjected to drought stress were used for gene expression analysis. Bars represents mean of three biological samples and error bars depicts the standard error and different alphabets represent statistically significant difference with P ≤ 0.05.

Quantitative real-time expression analysis (qRT-PCR) of transgenes was carried out in transgenic groundnut lines along with wild type plants in the T_3_ generation. The transgenic groundnut plants showed enhanced transcript levels compared to wild type under drought stress conditions. For example, *MuMYB96* showed a 2.88 to 4.38-fold increase in transcript abundance in multigene transgenic plants over wild type plants, whereas *MuWRKY3* transcript levels showed a 3.50 to 3.618-fold increase and 3.15 to 3.46-fold increase in transcript levels of *MuNAC4* gene. The overexpression of transcription factors resulted a significant increase in transcript level of downstream genes such as *KCS6*, and *KCR1, APX3* and *CSD1 LBD16* and *DBP* in transgenic plants over wild type plants under drought stress conditions (**Figure 3**).

### Morpho-physiological, growth and yield-related traits in multigene transgenic groundnut plants under drought stress

**Figure 4:**
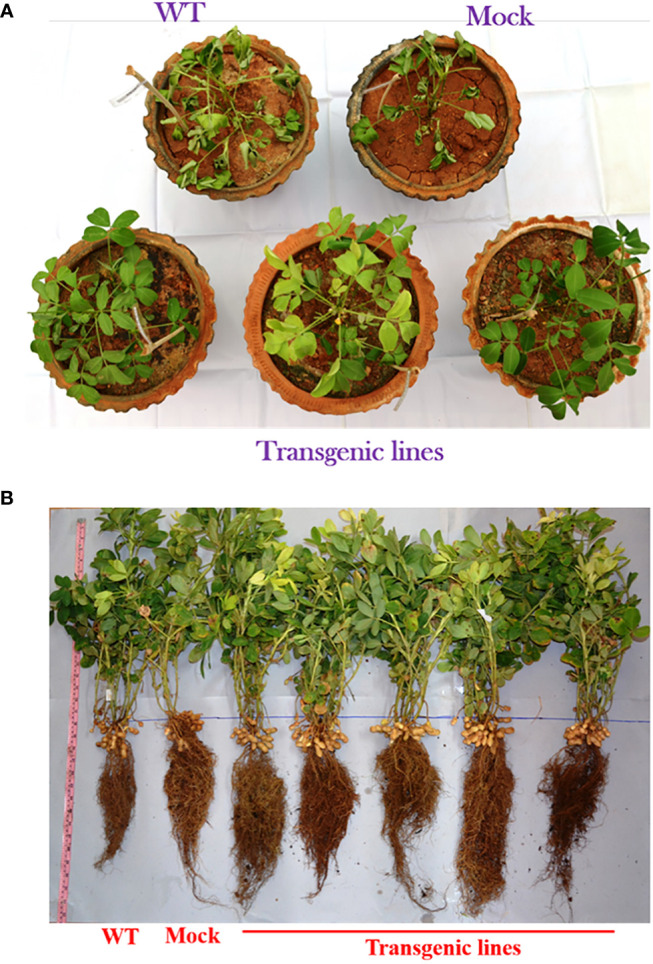
Response of groundnut transgenic plants to drought stress. **(A)**. Drought stress assay - Image showing the stay-green nature of multigene transgenic groundnut plants after 10 days of drought stress. Wild type (WT) and mock plants showed severe visible wilting symptoms whereas transgenic plants showed stay green nature under drought stress. **(B)** Phenotypic**-**trait analysis of multigene transgenic groundnut plants at harvest stage. Image showing profuse growth of lateral root density, shoot biomass and more pods in multigene transgenic plants compared to wild type and mock plants.

In T_3_ generation, 30-days-old multigene transgenic groundnut plants, wild type and mock plants were subjected to drought stress by withholding the water for 10-days. Drought stress resulted visible leaf wilting in both multigene transgenic plants, wild type and mock plants under drought stress, however, the symptoms appeared much earlier in wild type and mock plants, with significant phenotypic difference under drought stress. The transgenic plants showed mild wilting symptoms and remained green after ten days of drought stress imposition whereas wild type and mock plants showed severe visible wilting symptoms (**Figure 4A**).

Further, morphological parameters and yield related data was recorded for transgenic lines, wild type and mock plants after harvest and the multigene transgenic groundnut plants showed better growth and increased root length, more number of pegs and pods compared to wild type and mock plants (**Figure 4B**). In general, multigene transgenic lines, wild and mock plants showed significant difference in their growth. Multigene transgenic plants exhibited superior growth than the wild type and mock plants. Growth of lateral roots and overall root length of multigene transgenic groundnut lines significantly increased compared to wild type and mock plants. Consequently, pronounced increase in the root dry weights observed. Total number of pods was more in multigene transgenic lines compared to wild type and mock plants **(** **Table 1** **)**.

**Table 1:** Growth and yield related parameters in multigene transgenic groundnut plants, wild type and mock plants subjected to drought stress.

Genotype	Shoot length(cm/plant)	Root length(cm/plant)	Shoot dry weight(g/plant)	Root dry weight(g/plant)	No. of Pegs/plant	No. of Pods/plant	Pod dry weight/plant(g)
**Wild type**	34.33 ± 3.18^c^	26.40 ± 2.77^e^	19.50 ± 1.40^f^	1.47 ± 0.37^g^	21.33 ± 1.52^d^	16.00 ± 3.60^c^	13.32 ± 2.19^d^
**Mock**	35.00 ± 1.41^c^	24.93 ± 3.95^e^	18.60 ± 3.40^f^	2.32 ± 0.93^fg^	22.33 ± 4.72^d^	14.33 ± 1.52^c^	14.62 ± 3.74^d^
**TL2-2-2**	54.33 ± 4.58^a^	39.73 ± 3.05^abcd^	36.60 ± 1.03^ab^	3.81 ± 0.59^bcd^	40.33 ± 5.13^ab^	26.33 ± 4.16^ab^	26.04 ± 5.39^abc^
**TL6-2-1**	47.00 ± 1.81^b^	40.93 ± 2.87^abc^	25.89 ± 3.36^e^	4.58 ± 1.21^bc^	34.33 ± 4.16^bc^	27.00 ± 2.64^ab^	27.81 ± 2.19^ab^
**TL11-1-3**	53.33 ± 4.42^a^	39.70 ± 7.80^abcd^	31.01 ± 2.00^cd^	3.72 ± 0.44^cd^	42.00 ± 3.60^a^	28.33 ± 3.51^a^	25.05 ± 3.57^abc^
**TL19-1-3**	48.30 ± 2.12^b^	42.33 ± 6.20^ab^	41.43 ± 2.08^a^	5.07 ± 0.32^b^	40.66 ± 3.05^ab^	26.66 ± 4.72^ab^	29.06 ± 1.18^a^
**TL23-1-3**	48.00 ± 2.47^b^	41.00 ± 4.52^abc^	33.59 ± 4.34^bcd^	3.93 ± 0.90^bcd^	33.66 ± 0.57^c^	24.33 ± 1.52^ab^	25.84 ± 1.74^abc^
**TL29-1-1**	48.50 ± 2.48^b^	43.50 ± 4.17^ab^	35.10 ± 5.25^bc^	3.14 ± 0.32^def^	37.66 ± 1.52^abc^	23.66 ± 1.52^ab^	23.25 ± 2.52^bc^
**TL36-1-4**	45.76 ± 3.02^b^	46.33 ± 1.29^a^	28.583 ± 2.78^de^	6.44 ± 0.24^a^	34.33 ± 4.04^bc^	24.33 ± 3.05^ab^	24.98 ± 2.86^abc^
**TL40-2-4**	43.76 ± 1.91^b^	34.86 ± 1.05^cd^	32.34 ± 2.53^bcd^	2.42 ± 0.23^efg^	35.00 ± 4.00^bc^	23.33 ± 3.21^ab^	23.93 ± 3.38^abc^
**TL41-1-3**	48.33 ± 2.15^b^	33.60 ± 1.75^d^	34.38 ± 2.62^bc^	3.18 ± 1.55^def^	38.66 ± 6.50^abc^	24.66 ± 3.05^ab^	21.19 ± 4.92^c^
**TL42-2-3**	45.26 ± 2.75^b^	39.26 ± 1.49^bcd^	28.90 ± 0.98^de^	3.62 ± 0.44^cde^	36.00 ± 2.00^abc^	22.66 ± 2.08^b^	26.16 ± 1.49^abc^

The values are the mean of three biological experiments with triplicates ± SE. (*P ≤* 0.05).

**Figure 5:**
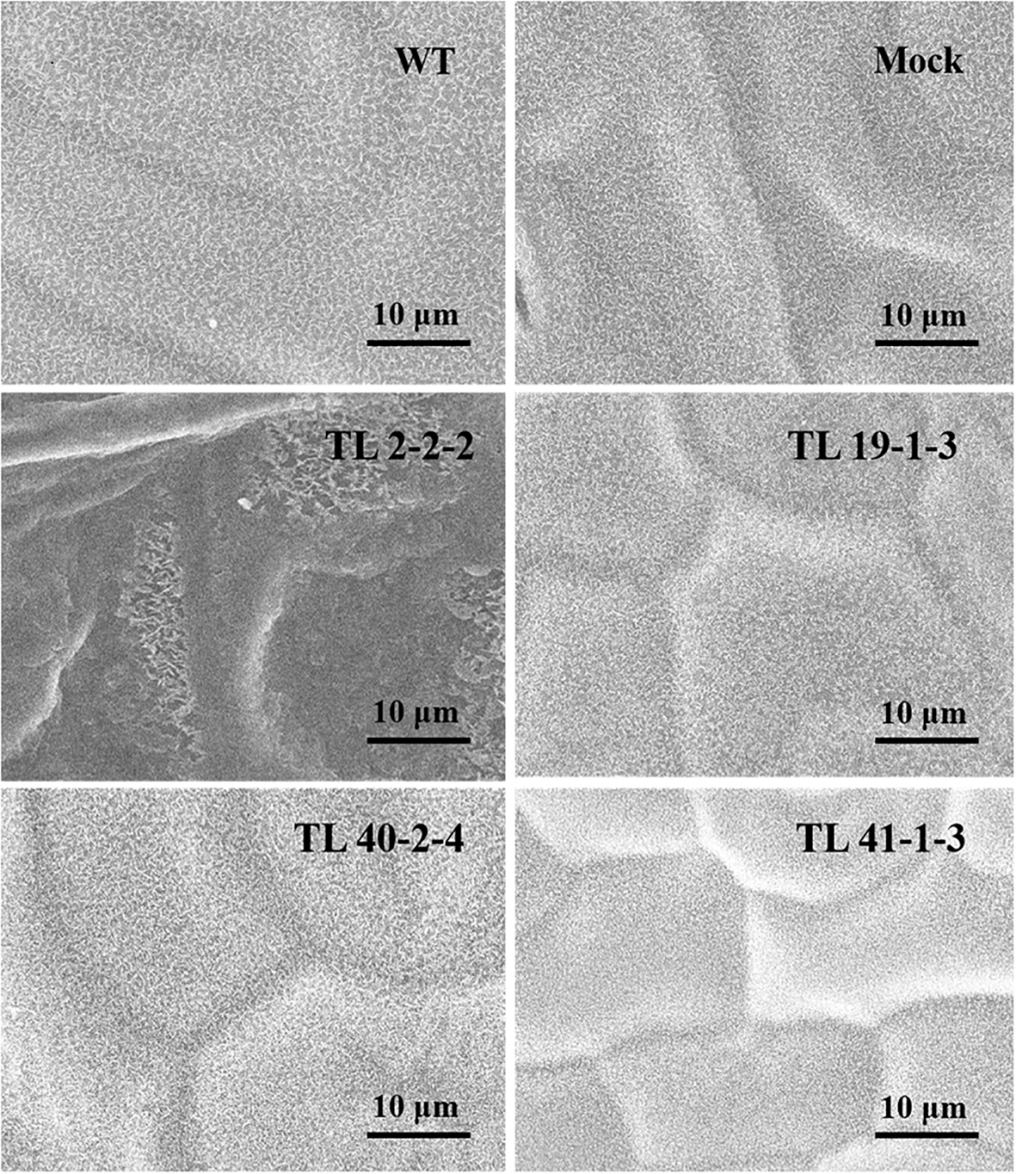
Scanning electron microscope (SEM) analysis of wax deposition on leaf surface in groundnut transgenics under drought stress: The image depicting the variation in deposition of cuticular waxes on the leaf surface (adaxial surface) of wild type (WT), mock and multigene transgenic groundnut lines (TL 2-2-2, TL 19-1-3, TL 40-2-4 and TL 41-1-3) under drought stress. The SEM images were taken at 10μm focal length.

**Figure 6:**
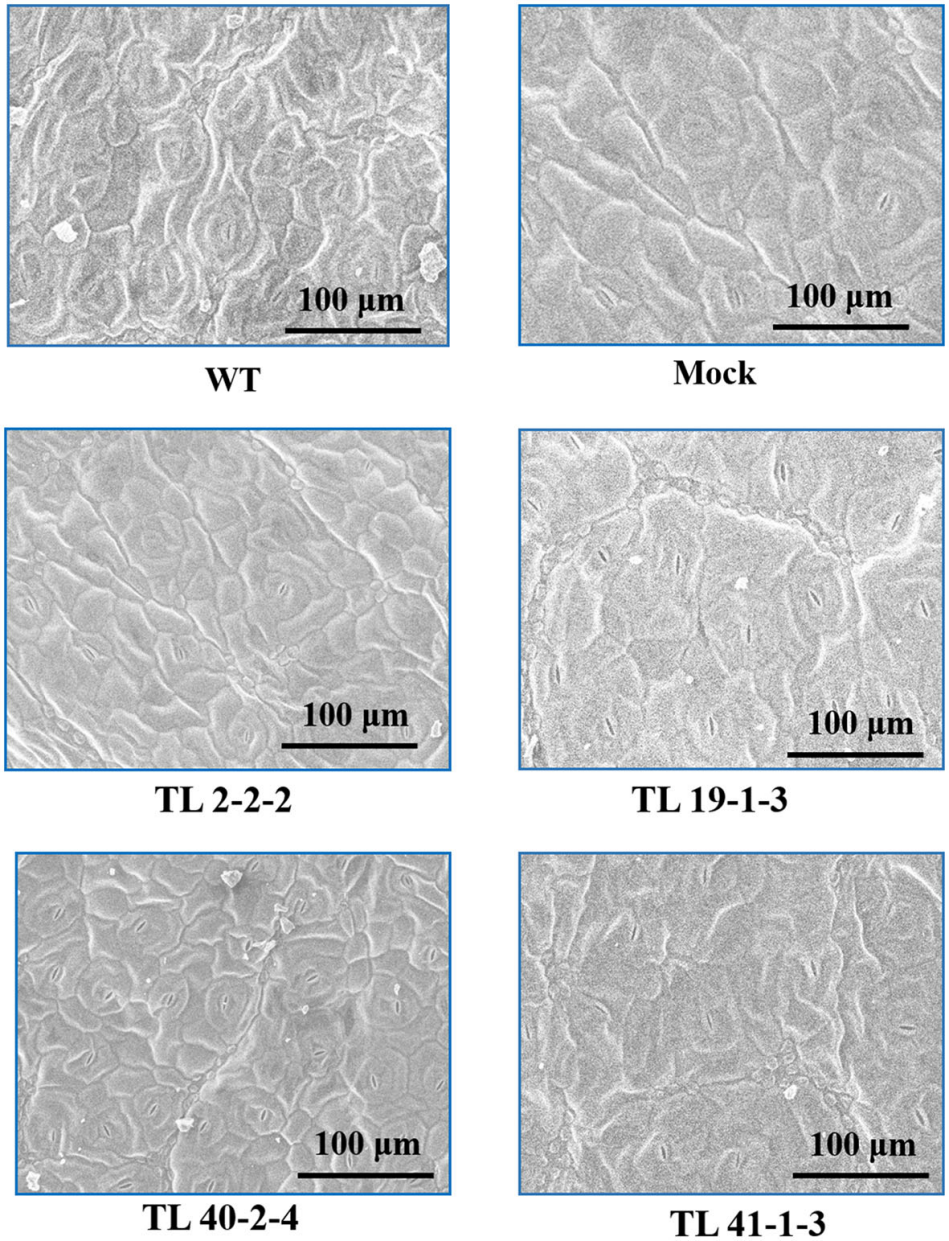
Scanning electron microscope (SEM) showing stomata number: The image depicting the variation in the stomata number on the leaf surface (adaxial surface) of wild type (WT), mock and multigene transgenic groundnut lines (TL 2-2-2, TL 19-1-3, TL 40-2-4 and TL 41-1-3) under drought stress. The SEM images were taken at 100μm focal length.

Variation in the epicuticular wax accumulation was observed between the multigene transgenic lines and wild type and mock plants. The transgenic plants showed a significant increase in the deposition of cuticular wax crystals over the wild type and mock samples. Leaf sample of TL2-2-2 transgenic line showed condensed cuticular crystals resulting in plaque-like deposits (**Figure 5**). The surface of the transgenic leaves (TL19-1-3, TL 40-2-4 and TL 41-1-3) exhibited dense wax crystals accumulation, whereas the mock and wild type plants have sparse wax accumulation In addition to the wax deposition, we observed variations in the stomatal number between multigene transgenic lines and wild type and mock plants. Wild type plants showed more number of stomata, whereas the transgenic plants showed less number of stomata (**Figure 6**).

**Figure 7:**
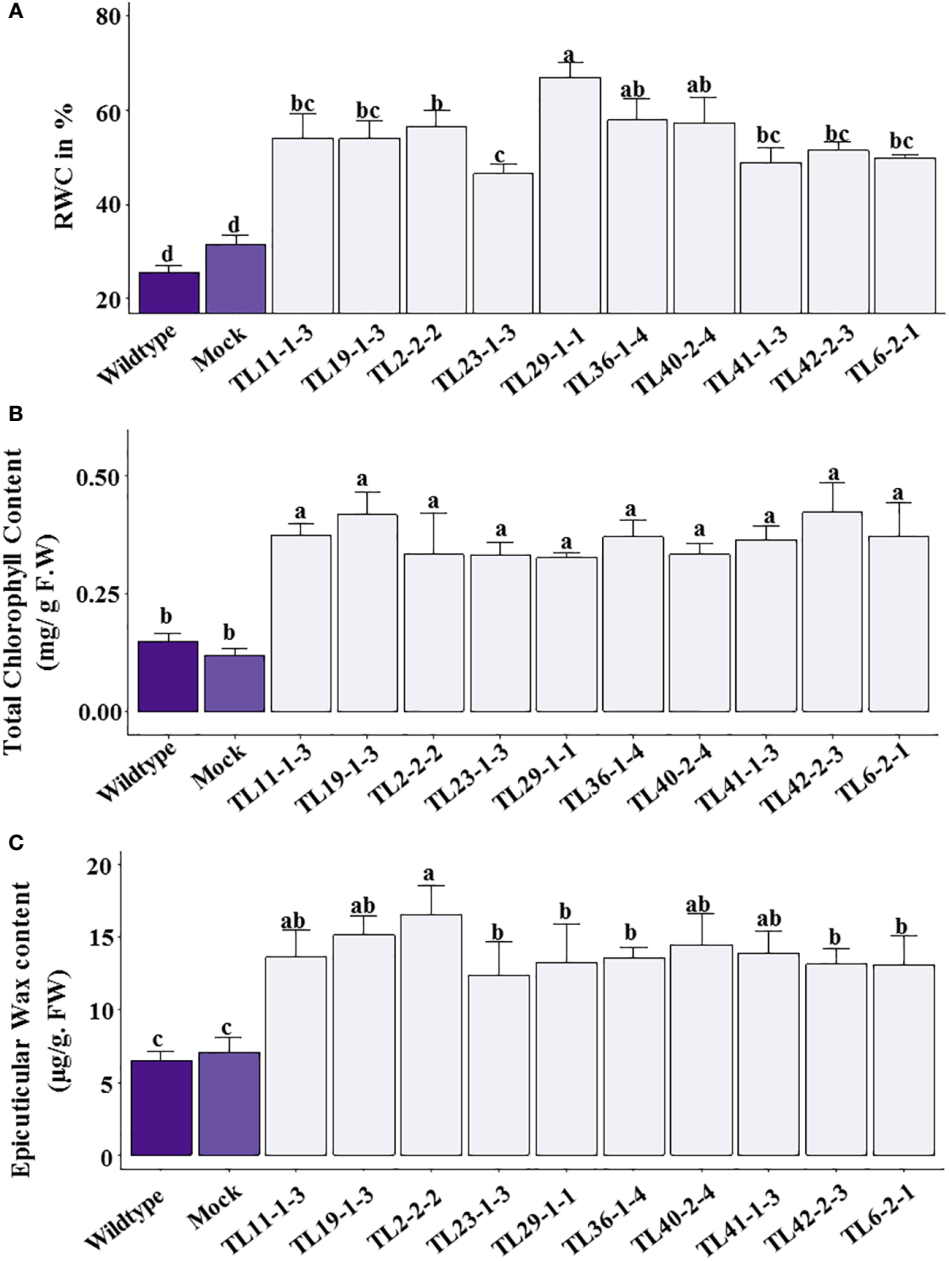
Physiological parameters in multigene transgenics lines, wild type and mock plants under drought stress: **(A)** Relative water content (% RWC), **(B)** Total chlorophyll content, **(C)** Epicuticular wax content. The values are mean of 3 biological replicates (n=3) and error bars denotes standard error. The alphabets on the error bars indicate significant variation (*p ≤* 0.05) between transgenic lines, wild type and mock plants.

The relative water content was significantly less in wild type compared to multigene transgenic lines under drought stress. The transgenic groundnut plants showed a range of 40.27 to 66.89% of relative water content. In contrast, wild type and mock plants showed 32.39% and 34.88%, respectively, demonstrating superior water retention capacity of transgenic plants than wild type plants under water stress conditions **(** **Figure 7A** **)**. Stress effect was more pronounced in wild type as evidenced by reduction in total chlorophyll content. The transgenic groundnut plants showed significantly higher chlorophyll content (0.26 to 0.42mg/g. FW) compared to wild-type (0.18mg/g. FW) and mock plants (0.218 mg/g. FW) (**Figure 7B**).

The multigene transgenic groundnut plants showed significantly higher epicuticular wax content ranging from 12.42 to 16.51µg/g F.W under drought stress conditions compared to wild type and mock plants (6.54 and 7.08µg/g F.W respectively), which is 2 to 2.5 folds lower than that of the transgenic groundnut plants (**Figure 7C**).

### Total soluble sugars, proline and malondialdehyde content in multigene transgenic groundnut plants under drought stress

**Figure 8:**
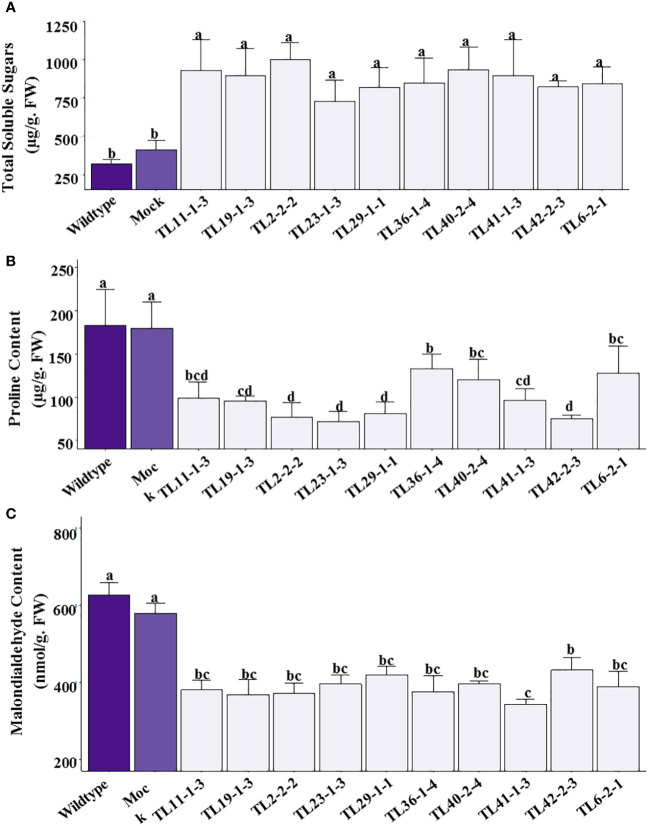
Osmolytes and Malondialdehyde content in multigene transgenic lines, wild type and mock plants under drought stress: **(A)**. Total soluble sugars, **(B)**. Free proline content, and **(C)**. Malondialdehyde content. The values are mean of three biological replicates (n=3) and error bars denotes standard error. The alphabets on the error bars indicates significant variation (*p ≤* 0.05) between transgenic lines, wild type and mock plants.

The transgenic groundnut plants showed significantly higher levels of total soluble sugars ranging from 760-997µg/g F.W under stress conditions. In comparison with transgenic plants, wild type and mock plants showed relatively lower levels of TSS, ranging 321.71 and 409.49µg/g F.W respectively (**Figure 8A**). The transgenic lines showed lower proline content ranging from 72-131µg/g F.W than wild type and mock plants which showed 184 and 179µg/g F.W, respectively (**Figure 8B**). There was significant decrease in proline content in multigene transgenic lines compared to wild type and mock plants under drought stress. The lower proline content could be due to better RWC and maintenance of high turgor potential which perhaps not sufficient enough to induce high proline content in multigene transgenic groundnut plants than the wild type and mock plants. Malondialdehyde, the end product of membrane lipid peroxidation, was quantified to assess the extent of oxidative damage caused by imposed drought stress. The multigene transgenic groundnut plants showed significantly lower levels of MDA (310.68-432.28nmol/g F.W) content than the wild type (627.85nmol/g F.W) and mock plants (491.57nmol/g F.W) (**Figure 8C**).

### Antioxidative efficacy in multigene transgenic groundnut plants under drought stress

**Figure 9:**
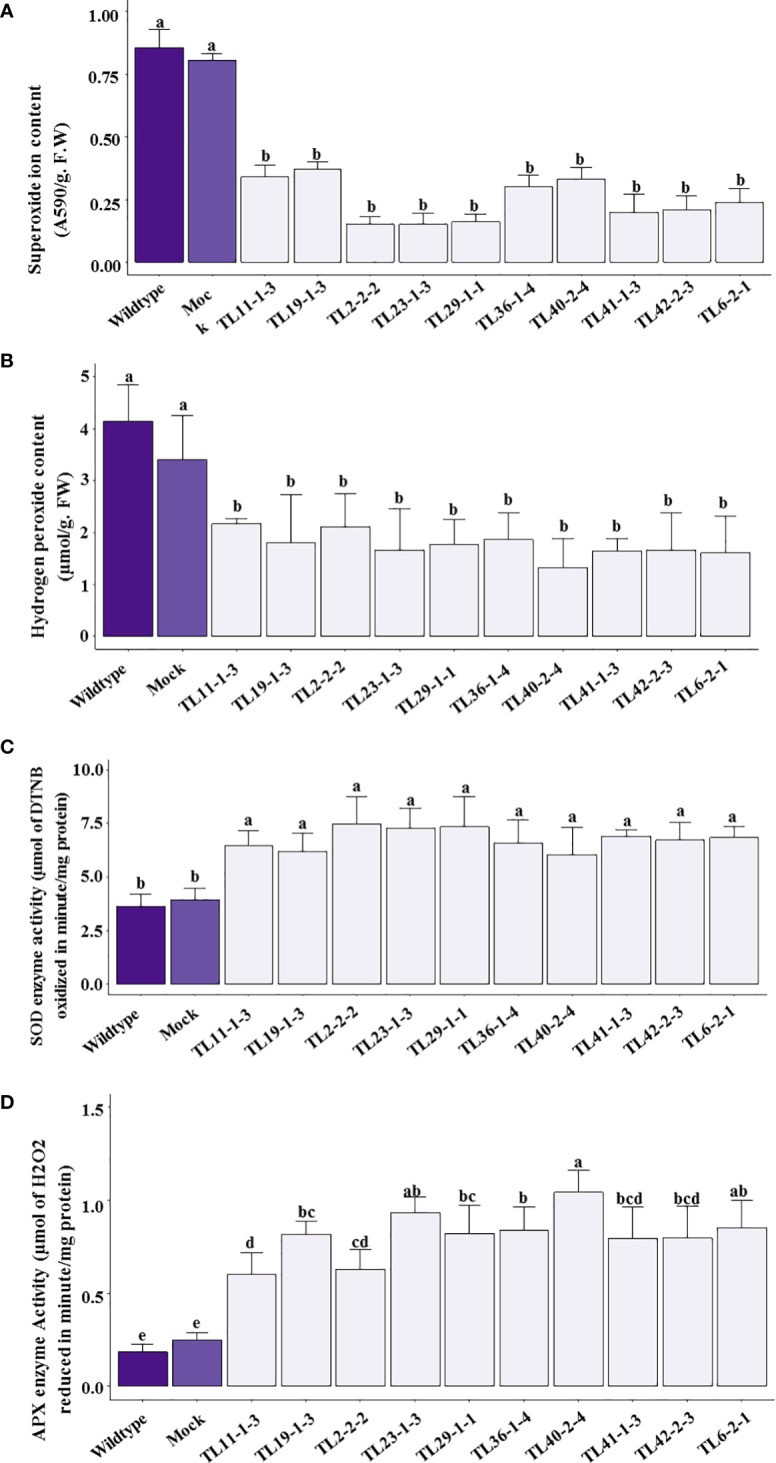
Reactive oxygen species (ROS) and anti-oxidative enzyme efficacy in multigene transgenic plants, wild type and mock plants under drought stress: **(A)**. superoxides, and **(B)**. hydrogen peroxide content), **(C)**. superoxide dismutase (SOD) and **(D)**. ascorbate peroxidase (APX) activity in wild type, mock and multigene transgenic groundnut plants under drought stress conditions. The values are mean of three biological replicates (n=3) and error bars denotes standard error. The alphabets on the error bars indicates significant variation (p<0.05) between transgenic lines, wild type and mock plants.

The wild type and mock plants showed a significant increase in superoxide production under drought stress conditions compared to transgenic plants. A two to four fold decrease in superoxide production was observed in multigene transgenic groundnut plants compared to wild type and mock plants (**Figure 9A**). Similarly, H_2_O_2_ production significantly increased in wild type and mock plants with 4.14μmol/g. F.W and 3.41μmol/g F.W of H_2_O_2_, respectively. The multigene transgenic plants showed a 2-3 fold lower levels of H_2_O_2_ production (**Figure 9B**).

The ROS was counter-attacked by antioxidative defense enzymes, such as SOD, and APX were measured in multigene transgenic groundnut plants, wild type, and mock plants under drought stress conditions. Results indicated significantly higher levels of SOD activity with a 2 to 2.2-fold increase in transgenic groundnut plants compared to wild type and mock plants (**Figure 9C**). In addition, the multigene transgenic plants exhibited APX activity with a range of 0.63-1.04µmol/mg protein/min, which is 3-5 folds higher than that of the wild type (0.18µmol/mg protein/min) and mock plants (0.24µmol/mg protein/min) (**Figure 9D**).

## Discussion

Drought stress affects several morpho-physiological, biochemical, and molecular changes in plants and often triggers the activation of signaling molecules and cascades involved in cellular responses (Sampaio et al., 2022). Several TFs as master regulators of gene expression were identified and reported to be controlling the mechanisms involved in drought stress tolerance (Erpen et al., 2018; Manna et al., 2021; Yoon et al., 2022). Several studies evidenced that overexpression of TF genes in crop plants resulted in enhanced drought stress tolerance (Yuan et al., 2020; Li et al., 2020). Drought stress resulted leaf wilting in multigene transgenic groundnut plants, wild type and transgenic lines. The visible wilting symptoms appeared much earlier in wild type and mock plants with reduced growth than transgenic plants under drought stress **(** **Figure 3** **).**

Multigene transgenic groundnut plants were developed by pyramiding *MuMYB96, MuWRKY3*, and *MuNAC4* genes through gateway cloning technology and evaluated for drought tolerance in comparison with wild type and mock plants. The multigene transgenic plants showed increased expression of the *MuMYB96* gene under drought conditions similar to that of the reports in *Camelina sativa* conferring enhanced drought tolerance (Lee et al., 2014). Cuticular wax forms the outer layer of areal parts and considered an early adaptive trait against water stress and protect the plants from harmful UV radiation and herbivory (Yeats and Rose, 2013; Tafolla-Arellano et al., 2018). Many researchers employed cuticular wax-related genes at the molecular level in conferring stress tolerance in crop plants (Lewandowska et al., 2020). The SEM analysis displayed dense deposition of wax crystals on the leaf surface of transgenic plants, whereas sparingly distributed wax crystals were observed in wild type plants (**Figure 4**). SEM results were supported by the wax content in transgenic plants under drought stress. The transgenic plants showed more than two-fold increase in cuticular wax content in the leaves of multigene transgenic plants than the wild type. *MuMYB96* transcript levels were significantly increased in multigene transgenic plants and also resulted in the overexpression of its downstream target genes *KCS6* and *KCR1*, supporting their role increased cuticular wax accumulation. Earlier drought induced expression of *MYB96* and its downstream genes *KCS6* and *KCR1* were reported in response to drought stress (Lee et al., 2016; Zhang et al., 2019; Lewandowska et al., 2020; Ahmad et al., 2021; Huang et al., 2022) (**Figure 8**).

Drought stress adversely affects plant-water relations, resulting in reduced cell turgor, stomata closure, restricted gas exchange, and photosynthetic machinery (Kheradmand et al., 2014; Kosar et al., 2015). Therefore, the stability of chlorophylls under water deficit conditions is considered a good criterion for drought tolerance (Arunyanark et al., 2008; Ahmed et al., 2020). In the present study, maximum retention of relative water content and chlorophylls was observed in the leaf tissues of transgenic groundnut plants compared to wild type and mock plants (**Figures 5A, B**). Furthermore, several previous investigations on overexpressing different regulatory and functional genes reported relatively higher chlorophyll content and RWC in transgenic groundnut plants, conferring improved drought tolerance (Bhatnagar-Mathur et al., 2014; Banavath et al., 2018; Lokesh et al., 2019; Venkatesh et al., 2019) and the results obtained in our study showed a similar trend suggesting the possible drought tolerant mechanism in groundnut transgenics.

Production of ROS (superoxides, peroxides, hydroxyl ions, etc.) is a common phenomenon in response to drought stress in plants, and hyper-accumulation of ROS is lethal (Gill and Tuteja, 2010; Laxa et al., 2019; Soares et al., 2019). Under drought stress, the multigene transgenic groundnut plants exhibited reduced levels of superoxide and hydrogen peroxide content; in contrast, an increased antioxidative enzyme (SOD and APX) activity was observed in transgenic plants over non-transgenic plants (**Figure 7**). These results were positively correlated with the qRTPCR analysis of *APX3* and *CSD1* genes, which showed 3 to 4-fold higher transcript levels in transgenic plants than in wild type plants (**Figure 8** **)**. Previous studies in various crop species reported enhanced expression of *MuWRKY3* gene and antioxidative genes (*SOD*, *CAT*, and *POD*) conferred oxidative defense in response to drought stress (Morita et al., 2011; Feng et al., 2014; Guo et al., 2018; Kiranmai et al., 2018) Low levels of malondialdehyde, a biomarker of lipid peroxidation in transgenic plants (**Figure 8C**), suggest that reduced oxidative damage in the plant cells under drought stress was possibly protected by the improved anti-oxidant machinery (Levine et al., 1994; Ramu et al., 2016; Kiranmai et al., 2018) Following previous studies, the overexpression of the *MuWRKY3* gene under drought stress improves the tolerance of transgenic groundnut (Kiranmai et al., 2018).

Osmoregulation, through the accumulation of osmolytes such as proline, sugars, betaines, polyols, etc., plays a crucial role in maintaining cell turgor under water stress (Verbruggen and Hermans, 2008; Blum, 2017). In the present investigation, we reported a significant accumulation of soluble sugars in multigene transgenic plants in correspondence with wild type plants under drought stress (**Figure 8A**). Zhang et al. (2017) reported a 17-24% increase in the total soluble sugar content in a drought-tolerant groundnut cultivar Shanhua 11 under drought-stress conditions. Overexpression of *PDH45*, *NAC4* and *WRKY3* in groundnut demonstrated hyperaccumulation of soluble sugars under drought stress (Manjulatha et al., 2014; Pandurangaiah et al., 2014; Kiranmai et al., 2018; Kokkanti et al., 2022). In contrast, multigene transgenic groundnut plants showed a lower proline content upon drought stress compared to wild type (**Figure 8B**). The lower level of proline could possibly be due to the maintenance of better RWC and partial cellular turgor potential in multigene transgenic plants. However, previous studies in groundnut upon co-expression of multiple genes (*Alfin1, PgHSF4*, and *PDH45*) showed increased proline content under moisture stress (Ramu et al., 2016).

A profuse root system has been considered an adaptive strategy to enhance water uptake under water-limited conditions (Basu et al., 2016). In the present study, the multigene transgenic lines showed increased root length (**Figure 3A**) and growth of lateral root volume than the wild type and mock plants. The current investigation also revealed the expression of root-associated genes such as *LBD16* and *DBP* with increased transcript levels in transgenic plants under drought-stress conditions (**Figure 8**). Previous reports demonstrated the role of *LBD16* and *DBP* genes in root initiation and lateral root development in different plant species under various abiotic stresses (Liu et al., 2018; Zhang et al., 2018). Overexpression of the *MuNAC4* transcription factor gene in groundnut resulted in increased root volume and biomass under drought stress (Pandurangaiah et al., 2014). In our study, *MuNAC4* gene expression and other TF genes possibly contribute to improved root architecture in multigene transgenic groundnut plants. In addition, transcript levels of *MuNAC4* were found to be higher in transgenic plants than in wild type plants under drought stress conditions. These results are in concomitant with previous studies by Pandurangaiah et al. (2014). Several studies reported that overexpression of TF genes and regulation of genes involved in root trait development were proved to enhance drought stress tolerance in crop plants (Le et al., 2011; Chen et al., 2018; Figueroa et al., 2021).

Overexpression of TFs, *MuMYB96, MuWRKY3*, and *MuNAC4* contributed to improved physiological and biochemical traits, which resulted in delayed wilting, and stay-green nature of leaves under drought stress, and complete recovery rate after stress withdrawal (**Figure 3**). Overexpression of single transcription factor genes in groundnut plants conferred stress tolerance against drought stress; however, in this study stacking multiple genes showed enhanced tolerance levels compared to single gene transgenics (Pandurangaiah et al., 2014; Ramu et al., 2016; Kiranmai et al., 2018; Venkatesh et al., 2019). In addition to enhanced drought stress tolerance, better growth traits like shoot and root volume, and yield traits like pod number, and pod dry weight in transgenic plants compared to wild type and mock plants also observed (**Table 1**).

## Conclusions

The present study focused on developing transgenic groundnut plants by simultaneously expressing three regulatory genes, *MuMYB96, MuWRKY3*, and *MuNAC4*, to enhance drought tolerance. Expression of the *MuMYB96* gene in multigene transgenic groundnut plants exhibited increased epicuticular wax accumulation, thereby reducing non-stomatal water loss under water-limited conditions. Furthermore, improved water mining traits like root length contributed to maintaining cell turgor and stay-green in transgenic plants under drought stress due to the overexpression of *MuNAC4* gene in multigene transgenics. Furthermore, the transgenic plants displayed increased osmolyte accumulation, anti-oxidant enzyme activity, and detoxification of ROS, resulting in improved cellular level drought tolerance could be due to the expression of the *MuWRKY3* gene along with the other two other TF genes. In summary, improvement of superior water conservation, water mining, and cellular level tolerance traits in groundnut transgenics suggest the pyramiding of multiple TF genes for improving the manifold traits is a viable option to cope with the drought stress impact on crop plants with a limited yield penalty.

## Data availability statement

The original contributions presented in the study are included in the article/**Supplementary Materials**. Further inquiries can be directed to the corresponding author.

## Author contributions

CS conceptualized and designed the experiments. BV performed the research. AV, NuJ, and NJ performed data analysis. AA contributed bioinformatics annotation. AV, BR, KM, KK, and MP contributed vector construction. CS, BV, KM, and AV wrote the paper. All authors provided inputs to develop the manuscript. All authors contributed to the article and approved the submitted version.

## Funding

CSIR-SRF fellowship (No: 09/383(0051)/2016-EMR-I) and DBT (BT/PR.15503/AGR/02/913/2015).

## Acknowledgments

We acknowledge the DBT (BT/PR.15503/AGR/02/913/2015) and CSIR-SRF fellowship (09/383(0051)/2016-EMR-I) Government of India, New Delhi for financial support in the form of a research grant to CS and BV. We greatly acknowledge Late Prof. M. Udayakumar and Dr. Ramu S Vemanna, University of Agricultural Sciences, Bengaluru for providing vectors for multisite gateway technology. We deeply condole the sudden demise of Late Prof. M. Udayakumar and we know that his passing will not only leave a void in our research, but in the hearts of all those who knew him. Prof. Udayakumar will always remain within our hearts and we dedicate this article to Prof. Udayakumar.

## Conflict of interest

The authors declare that the research was conducted in the absence of any commercial or financial relationships that could be construed as a potential conflict of interest.

## Publisher’s note

All claims expressed in this article are solely those of the authors and do not necessarily represent those of their affiliated organizations, or those of the publisher, the editors and the reviewers. Any product that may be evaluated in this article, or claim that may be made by its manufacturer, is not guaranteed or endorsed by the publisher.
