# Supplementary material for: Co-expression of stress-responsive regulatory genes, MuNAC4, MuWRKY3 and MuMYB96 associated with resistant-traits improves drought adaptation in transgenic groundnut (Arachis hypogaea l.) plants
Source: Front Plant Sci. 2022 Nov 16;13:1055851. doi: 10.3389/fpls.2022.1055851 (PMC9709484; doi:10.3389/fpls.2022.1055851)
Supplement: Supplementary file 1 [file DataSheet_1.doc]

**SUPPLEMENTARY TABLES**

**Supplementary Table 1:** List of genes used in the genomic DNA PCR analysis with their corresponding primers and annealing temperature.

| **Gene** | **Primers** | **Tm** |
| --- | --- | --- |
| *MuMYB96* | F: 5’ ATG GGC AGA CCA CCT TGT TG 3’  R: 5’ TCA GAA CAT GCC CTC TTG ACC 3’ | 59.1^o^ C |
| *MuWRKY3* | F: 5’ ATG GCT TCT GCT GCC TCT GC 3’  R: 5’ TCA TGG ACC CAT TAG TAT TC 3’ | 58.2^o^ C |
| *MuNAC4* | F: 5’ ATG GGA GTT CCA GAG GAA G 3’  R: 5’ TCA ATT CCT GAA CCC GAA C 3’ | 53.1^o^ C |

**Supplementary Table 2: List of primers used in qRT-PCR analysis**

| **Gene** | **Primers** | **Length** |
| --- | --- | --- |
| *RTMuMYB96* | F: 5’ CAACAAGGGACAGTGGGAAAG 3’  R: 5’ GGCAAAGAGCTGGAAGATGAA 3’ | 21bp  21bp |
| *RTMuWRKY3* | F: 5’ CACCAAGCAGTCAAGGGAGAA 3’  R: 5’ GGCGCCGTCGAAGAATG 3’ | 21bp  17bp |
| *RTMuNAC4* | F: 5’ GTTCGCCGTCGTCGTCAT 3’  R: 5’ CCTTCTCCTCCTGCTGTTGCT 3’ | 18bp  21bp |
| *RTKCS6* | F: 5’ GTTCGGCAACACTTCGTCTTC 3’  R: 5’ ACCCACTCCCGAAAGCAATC 3’ | 21bp  20bp |
| *RTKCR1* | F: 5’ CGCCAAGTTCCCCAAAATC 3’  R: 5’ CACCAACGTCCAATCCTTCAA 3’ | 19bp  21bp |
| *RTAPX3* | F: 5’ ATGGCGAAAGAGGGAGTAGCA 3’  R: 5’ AGCATGAGAGGAGCGCAGTT 3’ | 21bp  20bp |
| *RTCSD1* | F: 5’ CTGGAAATCTTGCTGGCCTTA 3’  R: 5’ TTGAAATGCGGTCCAGTTGA 3’ | 21bp  20bp |
| *RTLBD16* | F: 5’ CTCAGAACAAGGGCCTGCAA 3’  R: 5’ CAACAGCCTCACAGCGATCA 3’ | 20bp  20bp |
| *RTDBP* | F: 5’ CCAAGATGCTGCCGAGAAG 3’  R: 5’ TTTTAACTGGCTCCACTG CTTTC 3’ | 19bp  23bp |

| **Gene name** | **Gene Sequence** |
| --- | --- |
| *MuMYB96* | atgggcagaccaccttgttgcgaaaagataggcgtgaaaaagggaccttggaccccagaagaagatattatactcgttagttacatccag  gagcatggtccaggaaactggagaagcgttccaacccatacaggattgagaagatgcagcaagtcttgcagacttaggtggactaacta  ccttagacctggaatcaagaggggtaacttcaccgagcacgaagaaaagactatcgttcacctccaggctttgttgggtaacagatgggct  gcaattgcttcttacctccctgagaggacagataacgatattaagaactactggaacacacatttgaagaagaagttgaagaagatcaacga  gagcggagaagaagataacgatggtgttagctcctctaacaccagctctcagaagaaccatcagagcaccaacaagggacagtgggaa  agaaggttgcagactgatattaacatggcaaagcaagcactctgcgaagctctttccttggataagccttcttcaacactttcatcttccagctc  tttgcctacaccagttattacacagcaaaacatcagaaacttctcatctgcattgctcgataggtgctatgatccttcctctagctcatctagca  ctacaaccaccaccacatctaacaccactaacccttacccaagcggagtttacgcaagctccgctgaaaacattgcaagactcttgcagga  tttcatgaaggataccccaaaggctttgaccctctcctcttcatcacctgtgtctgaaactggtcctcttacagcagctgttagcgaagagg  gaggtgaaggtttcgagcagtctttcttctctttcaactctatggatgaaacccagaacttgacccaagagacttctttcttccatgatcaag  tgatcaagcctgaaatcactatggatcaggatcatggtttgatttctcaaggatcattgtccctcttcgaaaagtggctcttcgatgagcaatc  acacgaaatggttggtatggcactcgcaggtcaagagggcatgttctga |
| *MuWRKY3* | atggcttctgctgcctctgcttcatttcctaccactgtatgcttcaataccaatacggttgatgaccgaaaacccagcttctttgagtttaaacc  acactgtacatcaaatatggctcctgcagaccctgacaaccatgcaagtgaaaaatctactcaaatagatggtcaaggaaaagctcaacct  tttgactcgtcaccattagtaaaaaatgagatggcagtcccttctaatgaattaagtctatcatcacctgttcaaatggttaactcaggagttaat  gcccgtgttgaaggtgatttggatgaactgaaccctaggagcaacataacaactgggcttccagcatcacaagttgacaatagaggtagtg  gactttccgttgcagctgagagagtatctgctgatggatacaactggagaaagtatgggcagaaacatgttaaaggaagtgaatttccacgc  agttactacaaatgtacacatcctaactgcgaagtcaagaaactatttgaacgctctcatgatgggcaaatcactgagataatttacaagggaa  cacatgatcacccaaaacctcaaccaaatcgccgttactcaactggaactatcattactatgcaagaagagagatctgataaagcttctttgac  tagccgagatgacagaggatccaatatatgtggccaggtgtctcatccatctgagcctgacagtactccagagctatcacctgcagctacaa  atgatggtgatcaagagggtacaggatttttgtcaaaccggaatagtgatgaggttgatgatgatgatcccttctcaaagcgaagaaaaatgg  agcttggaaatgctgacattactcctgtagttaagcccatcagggagccacgggttgttgtacaaactctgagtgaggttgatatattggatgatg  gttaccgctggcgcaagtatgggcagaaggtggtgagaggcaatcctaaccctaggagttattacaaatgcacaaacacggggtgccccg  ttagaaaacatgtggaaagggcatctcatgatccaaaagctgtgataaccacatatgaggggaaacacaatcatgatgtaccaactgcaa  ggaataatagttgccatgacattgcaggaccagtaagtgctgctgggggacagacaagaattaggccggaagaaagtgataccatcagc  cttgaccttggtatgggaattagccctgctgcggaagacacaccaagcagtcaagggagaatgatgctttccgaatttggggacagcca  agctcacaccggcaattccaatttcaagtttgtccattcttcgacggcgccagtgtactttggtgttctaaataacaactctaacccatatggctc  tagagaaaatccaagtgatagtacatctttaaaccgttctgcttatccctgcccccagaacatgggaagaatactaatgggtccatga |
| *MuNAC4* | atgggagttccagaggaagaccctctttcccagttgagtctacctcctggttttcgcttctaccccaccgacgaggagcttctcgttcagtatctc  tgccgcaaggtcgccggccaccatttctctctcccgatcattgctgaaattgatttgtacaagctcgacccatgggttcttccaagcaaggcgatt  ttcggggagaaagagtggtactttttcagccctcgtgacaggaaatacccaaacgggtctcgacccaacagagtagccgggtcgggttattgg  aaagccaccggaaccgacaagatcatcaccactgaaggtagaaaagttggcatgaaaaaagccctcgttttttacgttgggaaagctccaaaa  ggcaccaaaaccaattggatcatgcacgagtatcgcctccttgactcttcccgaaagacaactggcaccaagctggatgactgggttctgtgtc  gtatatataagaagaactcgagtgtacagaaggcagtgcagaacgacgtggtttcgagcagggaacacacacaatacagcaacggttcgccg  tcgtcgtcatcatcccatctggacgacgttctggaatcgctgccagcgatcgacgaacggtgctttgcgatgccacgtgtcaacacggtgcagc  aacagcaggaggagaaggtgaacgttcagaacctgggctcgagtgggttggtggattgggccaaccctgcggttctgagttcggtggctgattt  cgtttcggaaaataatcaagtggtgcaggaccatactcaggggatggtgaactacaacggctgcaatgacctttatgtccccaccttctgccacgt  ggactcctcgcttccgcaaaaggttgaggaagaggtgcaaagcggtgtcagaatccaaaacaataacccgtggtttcttcagaacgatttcacac  aggggtttcaaaaccctgtcgacacgtgtgggtttaagttcccggttcagccggtcgggttcgggttcaggaattga |

**Supplementary Table. 3:** Gene sequences of *MuMYB96, MuWRKY3* and *MuNAC4*
