# Supplementary material for: Co-expression of stress-responsive regulatory genes, MuNAC4, MuWRKY3 and MuMYB96 associated with resistant-traits improves drought adaptation in transgenic groundnut (Arachis hypogaea l.) plants
Source: Front Plant Sci. 2022 Nov 16;13:1055851. doi: 10.3389/fpls.2022.1055851 (PMC9709484; doi:10.3389/fpls.2022.1055851)
Supplement: Supplementary file 2 [file DataSheet_2.doc]

**
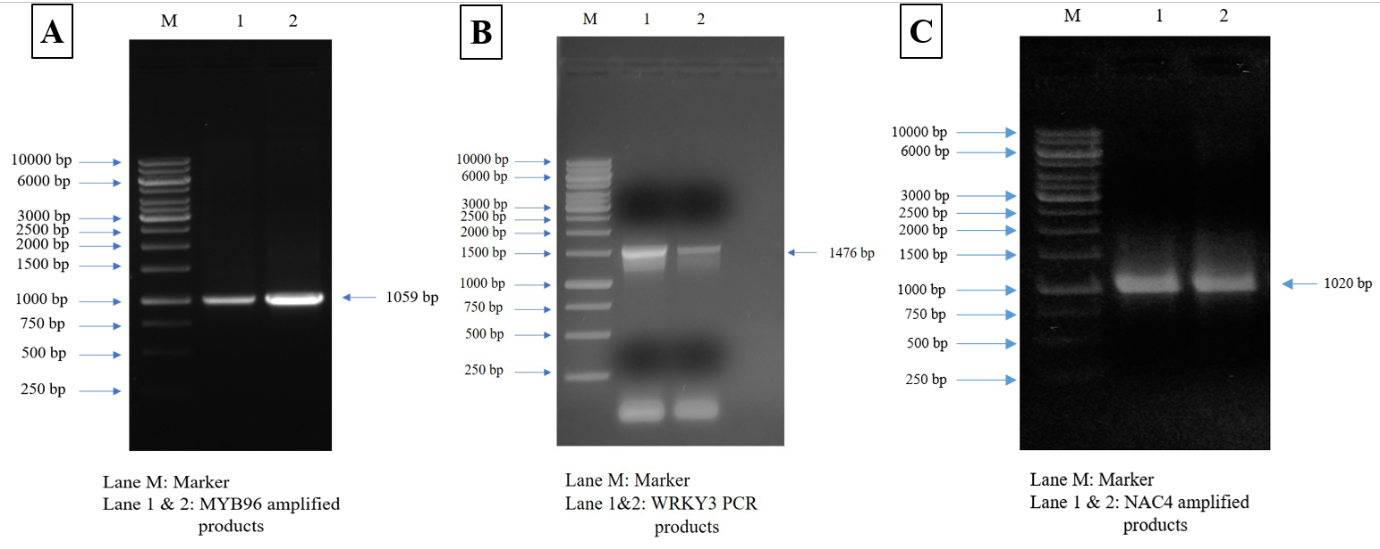
**

**Supplementary Figure 1**: **PCR amplification of three transcription factor genes from cDNA isolated from the horse gram leaves exposed drought stress. a).** Amplification of *MuMYB96* showing corresponding DNA band at 1059bps, **b).** *MuWRKY3* corresponding DNA band showing amplification at 1476bps, and **c).** *MuNAC4* amplification product showing DNA band at 1020bps. M; 1 kb ladder.

IM 1.1 + *MuMYB96* TA

pB4NU + *MuNAC4* TA

pRT100 + *MuWRKY3* TA


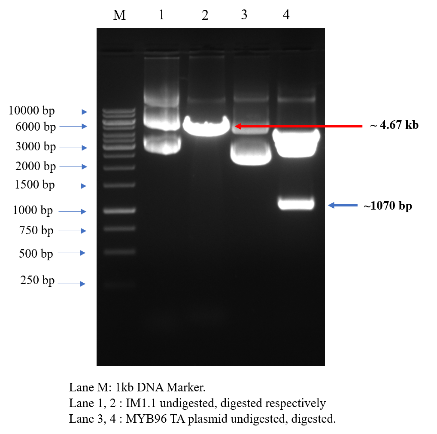

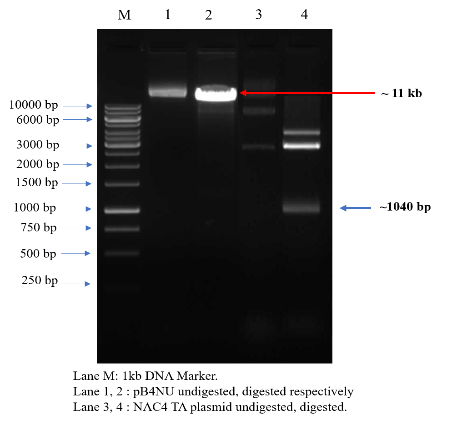

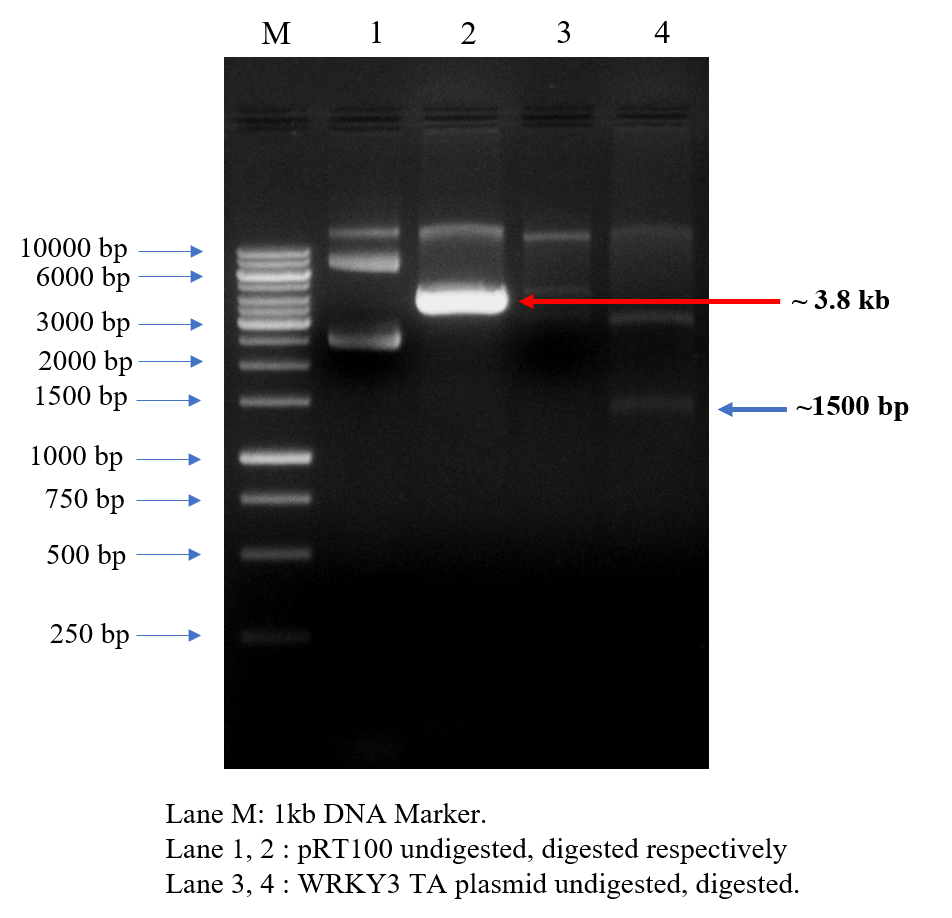


*Rbcs* P

*MuMYB96*

*Rbcs T*

XbaI

SacI

*2x35S* P

*MuWRKY3*

*PolyA* T

XhoI

XbaI

*Ubi P*

*MuNAC4*

*nos T*

BamHI

KpnI

**Supplementary figure 1a:** Cloning genes in to expression vectors:

*MuMYB96-*TA plasmid was restriction digested with XbaI and SacI to release gene fragment and same enzymes used to linearize IM1.1 vector. Both the fragments ligated to form the expression cassette. XhoI and XbaI enzymes were used to *MuWRKY3-*TA and pRT100 to prepare *WRKY3* expression cassette. *MuNAC4* expression cassette developed by digsting*MuNAC4-*TA & pB4NU with BamHI and KpnI enzymes.

**
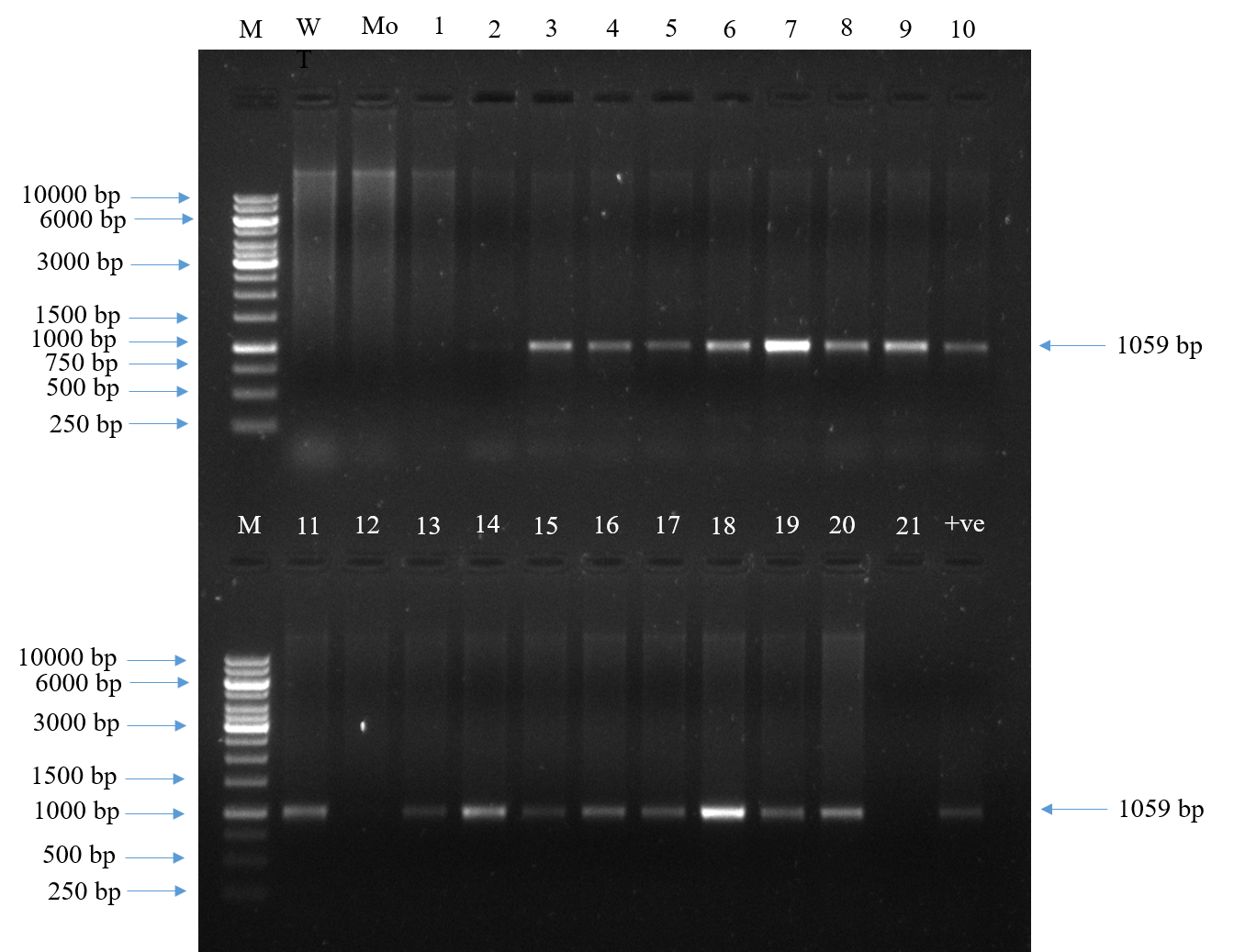

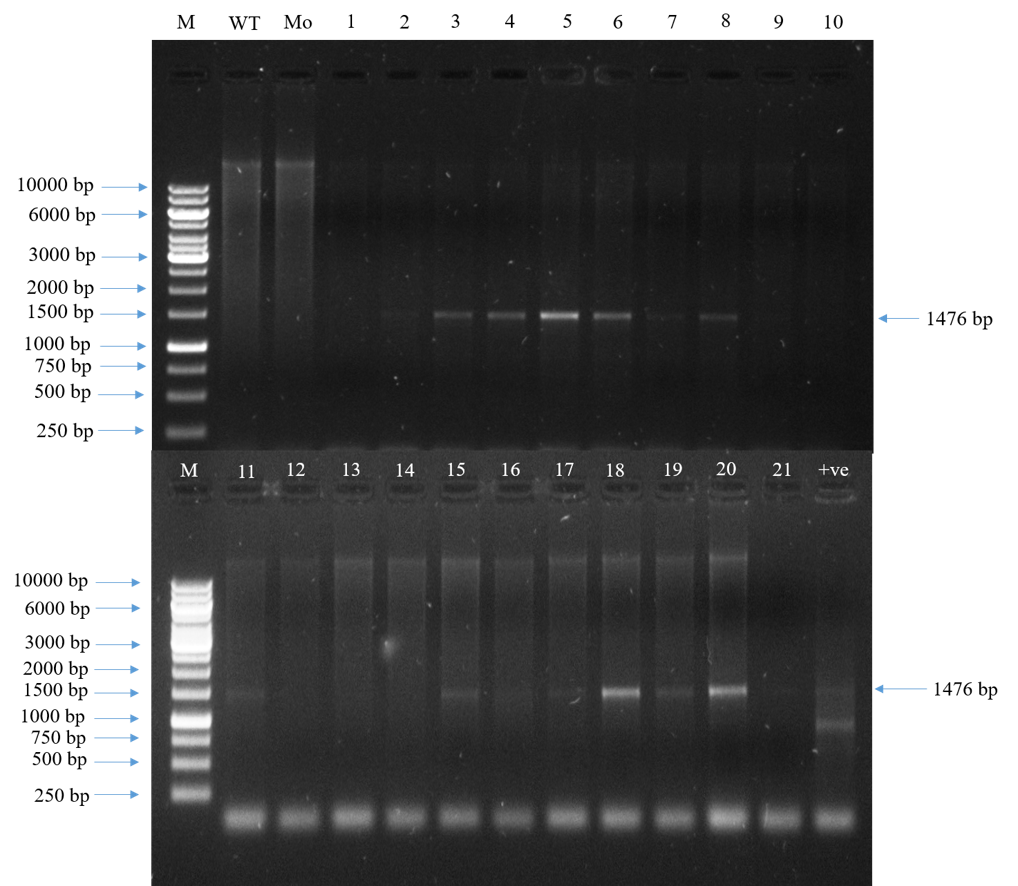

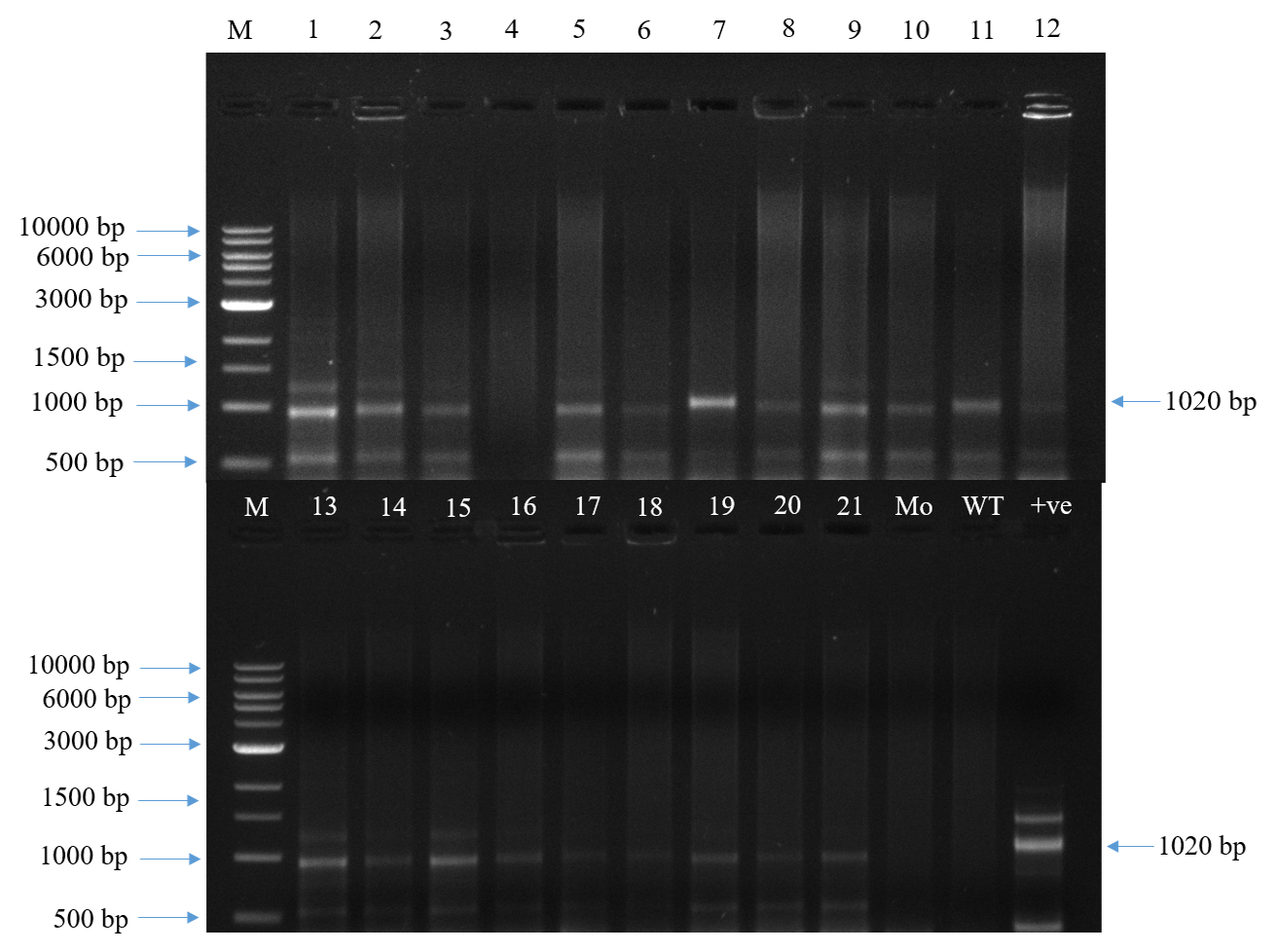
**

**a).**

**b).**

**c).**

**Supplementary Figure 2:** **Molecular characterization of transgenes (*MuMYB96:MuWRKY3:MuNAC4*) from multigene groundnut transgenics using genomic DNA as template**. **a).** Transgenic plants showing the amplification bands at 1059 bp confirming the presence of *MuMYB96* gene, **b).** Confirmation of *MuWRKY3* gene in transgenic lines showing corresponding DNA band at 1476 bp, **c).** Amplification bands at 1020 bp confirming the presence of *MuNAC4* in transgenic groundnut plants. Where **M:** gene marker (1 kb ladder), **WT:** wildtype, **Mo:** mock plants (transgenic plants without transgenes), **1-21:** multigene transgenic plants, **+ve:** plasmid control.

**C).**

**Advancement multigene groundnut transgenic plants from T_0_ through T_3_**

56 transgenic lines were raised in T_0_ generation and seeds were collected

A total of 41 putative transgenic lines were identified in T_1_ generation based on kanamycin screening

In T_1_ generation, 24 lines were PCR positive for marker gene (*nptII*) and transgenes (*MuMYB96, MuWRKY3* and *MuNAC4*) were advanced to T_2_ generation

In T_2_ generation, 21 lines showed normal growth on Kn^+^ medium and 17 lines were PCR positive for all the three transgenes

Among 17 lines, 13 lines were advanced to T_3_ generation based on the

drought stress indices

In T_3_ generation all the 13 lines showed Kn^+^ resistance and showed the presence of transgenes

Out of 13 multigene transgenic lines in T_3_ generation, 10 lines were evaluated for drought stress tolerance through physiological, biochemical and morphological traits in comparison with non-transformed plants

**Supplementary Figure 3: Flow chart showing the events of transgenic advancement from T_0_ generation to T_3_ generation.** The transgenic plants were selected based on kanamycin screening and stable integration of genes and were advanced to the next generation.
